# The correlation of muscle quantity and quality between all vertebra levels and level L3, measured with CT: An exploratory study

**DOI:** 10.3389/fnut.2023.1148809

**Published:** 2023-02-23

**Authors:** Jona Van den Broeck, Martine J. Sealy, Carola Brussaard, Jasmijn Kooijman, Harriët Jager-Wittenaar, Aldo Scafoglieri

**Affiliations:** ^1^Experimental Anatomy Research Group, Department of Physiotherapy, Human Physiology and Anatomy, Faculty of Physical Education and Physiotherapy, Vrije Universiteit Brussel, Brussels, Belgium; ^2^Research Group Healthy Ageing, Allied Health Care and Nursing, Hanze University of Applied Sciences, Groningen, Netherlands; ^3^Department of Radiology, Universitair Ziekenhuis Brussel, Brussels, Belgium; ^4^Department of Medical Imaging and Radiation Therapy, Hanze University of Applied Sciences, Groningen, Netherlands; ^5^Department of Oral and Maxillofacial Surgery, University of Groningen, University Medical Center Groningen, Groningen, Netherlands

**Keywords:** correlation, muscle quantity, muscle quality, cancer, computed tomography, SMA, SMI, MRA

## Abstract

**Introduction:**

In patients with cancer, low muscle mass has been associated with a higher risk of fatigue, poorer treatment outcomes, and mortality. To determine body composition with computed tomography (CT), measuring the muscle quantity at the level of lumbar 3 (L3) is suggested. However, in patients with cancer, CT imaging of the L3 level is not always available. Thus far, little is known about the extent to which other vertebra levels could be useful for measuring muscle status. In this study, we aimed to assess the correlation of the muscle quantity and quality between any vertebra level and L3 level in patients with various tumor localizations.

**Methods:**

Two hundred-twenty Positron Emission Tomography (PET)-CT images of patients with four different tumor localizations were included: 1. head and neck (*n* = 34), 2. esophagus (*n* = 45), 3. lung (*n* = 54), and 4. melanoma (*n* = 87). From the whole body scan, 24 slices were used, i.e., one for each vertebra level. Two examiners contoured the muscles independently. After contouring, muscle quantity was estimated by calculating skeletal muscle area (SMA) and skeletal muscle index (SMI). Muscle quality was assessed by calculating muscle radiation attenuation (MRA). Pearson correlation coefficient was used to determine whether the other vertebra levels correlate with L3 level.

**Results:**

For SMA, strong correlations were found between C1–C3 and L3, and C7–L5 and L3 (*r* = 0.72–0.95). For SMI, strong correlations were found between the levels C1–C2, C7–T5, T7–L5, and L3 (*r* = 0.70–0.93), respectively. For MRA, strong correlations were found between T1–L5 and L3 (*r* = 0.71–0.95).

**Discussion:**

For muscle quantity, the correlations between the cervical, thoracic, and lumbar levels are good, except for the cervical levels in patients with esophageal cancer. For muscle quality, the correlations between the other levels and L3 are good, except for the cervical levels in patients with melanoma. If visualization of L3 on the CT scan is absent, the other thoracic and lumbar vertebra levels could serve as a proxy to measure muscle quantity and quality in patients with head and neck, esophageal, lung cancer, and melanoma, whereas the cervical levels may be less reliable as a proxy in some patient groups.

## 1. Introduction

Malnutrition and sarcopenia are highly prevalent in patients with cancer (1, 2). These nutrition (-related) disorders are linked to a combination of reduced food intake, loss of muscle quantity and quality, with or without the loss of fat mass, and poor physical performance (3–5). Previous studies show that low muscle quantity and quality are firmly associated with poorer clinical outcomes in patients with cancer (2, 6, 7). Patients with cancer with low muscle quantity and quality also have a higher risk of cancer-induced fatigue, lower quality of life, and mortality (1, 8, 9). When chemotherapy treatment is given to patients with cancer, it is often based on the body surface area (BSA). However, the BSA does not sufficiently take into account the interpersonal variations of body composition in patients with cancer, which could result in a higher risk of toxicity and incomplete treatment (7, 10–12). Therefore, in patients with cancer, it is important to measure muscle quantity (6). In addition, measuring muscle quantity is also an important part of evaluation of the nutritional status of the patient (5, 13).

To define muscle quantity, skeletal muscle cross-sectional area (SMA) and skeletal muscle index (SMI) can be measured with computed tomography (CT), a gold standard for body composition measurement (1). The SMI shows the relative muscle quantity, as it is corrected for height (SMI = SMA/height^2^) (2). For this purpose, the third lumbar vertebra level (L3) is used, as the SMA correlates strongly with the muscle mass of the whole body (13, 14). It has also been shown that the levels above and below (±10 cm) L3 correlate well with the muscle mass of the entire body (14). However, a whole body CT scan is not always available in patients with cancer (7, 15). When the lumbar levels are not included in the CT scan image, for example in patients with head and neck cancer (7), it is unclear which vertebra levels can be used to estimate whole body muscle mass. In earlier studies in patients with head and neck cancer, the cervical level 3 (C3) and thoracic level 4 (T4) were used to measure muscles, and these levels showed a good correlation with L3 (7).

According to the European Working Group on Sarcopenia in Older People, muscle quality can be measured by muscle radiation attenuation (MRA), using CT images (5). Muscle quality is defined as muscle strength or power per unit of muscle mass and is closely intertwined with muscle strength (16). Intermuscular adipose tissue is an important factor underpinning muscle quality and also predicts muscle function (17). The intermuscular adipose tissue is located within the muscle, under the fascia, and encompasses intramuscular fat and low-density lean tissue (18). Muscle radiation attenuation closely correlates with direct measurements of muscle lipid content and therefore determines infiltration of fat into the muscle (19–21).

In addition, limited evidence regarding the correlation of muscle quantity and quality between vertebra levels other than L3 and the L3 level is available (22). As a first step in the search for which other vertebra levels, other than L3, could be used to determine whole body muscle mass, we aimed to examine the correlations between all vertebra levels with L3 for muscle quantity and quality in a sample of patients with various tumor localizations.

## 2. Materials and methods

### 2.1. Participants

Positron Emission Tomography CT (PET-CT) images of the participants were retrospectively extracted from medical records of the Radiology department of the University Hospital Brussels from December 2019 until February 2021. Patients aged ≥18 years with any of the following four localizations of newly diagnosed tumors were included: 1. head and neck cancer, 2. esophageal cancer, 3. lung cancer, and 4. melanoma. We excluded participants receiving treatment for current cancer at the time of the PET-CT scan and who had a previous diagnosis of cancer at another tumor localization. PET-CT images were included if they were performed between 2014 and February 2021. Sex, age (years), body weight (kg), body height (m), body mass index (BMI; kg/m^2^), cancer stage, and Charlson Comorbidity Index (CCI) (23) were retrieved from the patients’ medical chart.

### 2.2. Scanning procedure

The PET-CT images were performed with three different CT devices: Philips GEMINI TF TOF 64, SIEMENS Biograph20, and SIEMENS Biograph128. The patients were scanned helically with a slice thickness of 2 mm and 120 kilovoltage peak (kVp). An intravenous iodinated contrast agent was used in all patients, except for 15% of the patients with a contra-indication for this contrast: i.e., the contrast was recently applied for another CT procedure in the short term or the patients had problems with their kidneys.

### 2.3. Image analysis

**FIGURE 1:**
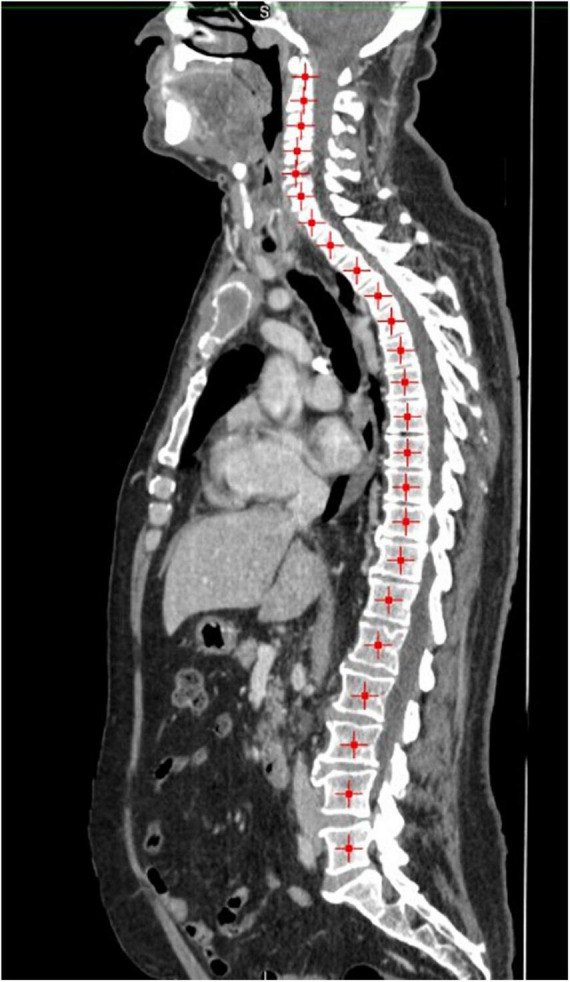
Twenty-four manually selected points on the vertebral column.

**FIGURE 2:**
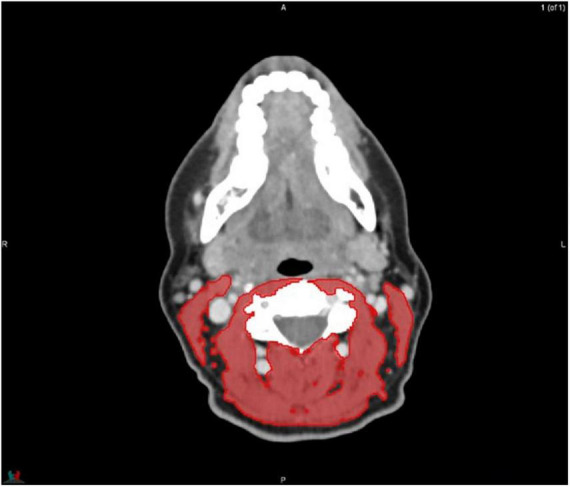
Contouring of the muscles at cervical level 3.

**FIGURE 3:**
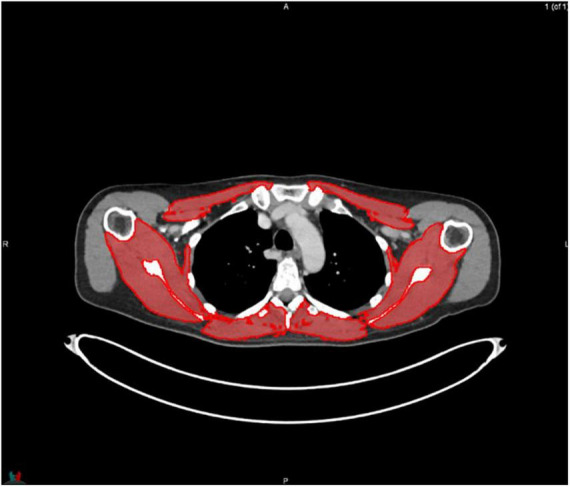
Contouring of the muscles at thoracic level 4.

**FIGURE 4:**
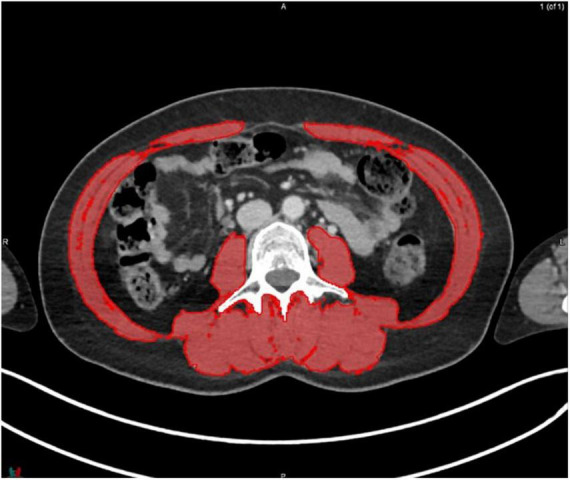
Contouring of the muscles at lumbar level 3.

MIM software (Version 7.0.1) was used to process the images. The whole-body scan was uploaded, after which 24 points were selected manually in the sagittal plane by a researcher (JV), as shown in Figure 1. The researcher selected images based on the center of each vertebral body. With the Launch Workflow procedure, 24 transverse slices were taken at the chosen points. This procedure allows a consistent and precise image selection. The slices were used to contour the muscles, as shown in Figures 2–4. Trunk muscles included in the contouring were the psoas, paraspinal, and abdominal wall muscles (2). In total, 12 examiners participated in this study to contour the muscles. In each slice, the muscles were measured twice. The two measurements were each performed by a different examiner, i.e., students from the Medical Imaging and Radiotherapeutic Techniques training program of Hanze University of Applied Sciences, Groningen, Netherlands, who were trained in muscle anatomy by an expert. During the process, the contouring by the examiners was regularly checked by this expert. Examiners were blinded to each other’s measurements and the characteristics of the patient. To contour the muscles, the Hounsfield units (HU) were set at a range lock between −29 and 150 HU (24). After contouring, SMA and MRA were calculated with the MIM software program. To calculate SMI, SMA was corrected for squared height in meters (cm^2^/m^2^). SMA was recorded in cm^2^, SMI in cm^2^/m^2^, and MRA in HU.

### 2.4. Statistical analysis

IBM SPSS statistics 26 was used to perform the statistical analyses. A Shapiro–Wilk test was performed to examine the normality of the distribution of the data. Normally distributed data are presented as mean and standard deviation (SD). Not normally distributed data are presented as median and interquartile range. An intraclass correlation coefficient (ICC) was calculated to analyze interrater reliability. When the data were not normally distributed, bootstrapping was applied to indicate whether the ICC was likely to be affected by the distribution of the data. With a high bootstrapping value (≥0.90), the ICC was not likely to be effected by the distribution of the data and the ICC value was accepted. When ICC values ranged between 0.0 and 0.20, the reliability was considered as slight, between 0.21 and 0.50 as poor, between 0.51 and 0.75 as moderate, between 0.76 and 0.90 as good, and 0.91 or above as excellent (25).

Next, Pearson correlation coefficients were calculated to assess whether the other levels of the spine correlated with the L3 level. Therefore, we took the average value of both examiners for each vertebra level. Finally, Pearson correlation coefficients were determined to analyze the correlation between all other levels with the L3 level, to study whether the tumor localization influenced the reliability. A Pearson correlation coefficient ≥0.70 is considered a strong correlation (25). *Post hoc* power analyses, using G*Power, were performed to analyze the power for each correlation. Power of 0.80 or higher was considered sufficient. For all analyses, the level of significance was set at *p* < 0.05.

## 3. Results

In total, 220 patients, including 34 patients with head and neck cancer, 45 with esophageal cancer, 54 with lung cancer, and 87 with melanoma, were included. Characteristics of the included patients are shown in Table 1. The descriptive data for SMA, SMI, and MRA at all vertebral levels are shown in Table 2.

**TABLE 1:** Characteristics of included patients.

		Total (*n* = 220)	Women (*n* = 64)	Men (*n* = 156)
Cancer type	Head and neck	34 (15%)	18 (28%)	16 (10%)
	Esophageal	45 (20%)	6 (10%)	39 (25%)
	Lung	54 (25%)	18 (28%)	36 (23%)
	Melanoma	87 (40%)	22 (34%)	65 (42%)
Age (years)		65.1 ± 10.9	63.8 ± 11.72	65.6 ± 10.6
Weight (kg)		74.5 [63.0–85.8]	62.0 [55.3–77.3]	78.0 [68.0–90.0]
Height (m)		1.71 ± 0.10	1.62 ± 0.06	1.74 ± 0.08
BMI (kg/m^2^)		25.4 [22.4–29.0]	23.8 [20.4–29.2]	25.7 [23.2–29.0]
Cancer stage	Grade 1	25 (11%)	6 (9%)	19 (12%)
	Grade 2	41 (19%)	13 (20%)	28 (18%)
	Grade 3	70 (32%)	24 (38%)	46 (29%)
	Grade 4	59 (27%)	8 (13%)	51 (33%)
	Unknown	25 (11%)	13 (20%)	12 (8%)
CCI		3 [2–6]	3 [2–6]	3 [2–6]

Data are presented as mean ± SD or as median [interquartile range].

CCI, Charlsons’ Comorbidity Index.

**TABLE 2:** Results for muscle quantity and quality at all vertebra levels.

	Total (*n* = 220)		
	SMA (cm^2^)	SMI (cm^2^/m^2^)	MRA (HU)
C1	38.3 [32.3–45.1]	13.3 [11.4–14.9]	43.2 [37.9–49.0]
C2	38.1 [31.6–43.6]	12.9 [11.1–14.9]	42.7 ± 8.6
C3	42.7 [35.1–48.9]	14.4 [12.4–16.5]	45.7 ± 12.9
C4	50.9 [41.5–62.5]	17.1 [14.7–20.9]	44.9 ± 10.6
C5	69.9 [54.1–102.2]	22.9 [18.2–34.9]	40.0 ± 12.3
C6	105.7 [74.0–147.2]	36.8 [25.8–51.3]	34.4 ± 9.1
C7	142.3 [115.5–181.0]	49.6 ± 14.7	35.8 [30.0–40.9]
T1	168.1 ± 46.7	56.1 [47.3–65.1]	36.8 ± 7.3
T2	178.4 [145.6–217.4]	61.3 [51.4–71.0]	38.7 ± 7.7
T3	186.7 ± 45.9	62.4 [53.6–72.6]	38.5 ± 7.3
T4	176.6 [147.3–212.4]	59.6 [52.1–70.0]	38.1 [32.7–42.1]
T5	158.1 [131.9–192.5]	54.5 [47.1–64.2]	36.2 [31.2–40.9]
T6	132.1 [111.5–160.2]	45.5 [39.5–53.3]	32.8 ± 8.2
T7	111.8 [90.1–140.4]	37.3 [32.7–44.4]	30.6 [25.4–35.9]
T8	93.3 [74.9–116.9]	32.3 [26.4–38.5]	28.7 [23.9–34.8]
T9	80.3 [65.3–98.0]	27.1 [22.7–32.1]	27.3 ± 8.9
T10	73.3 [59.6–88.4]	24.5 [20.5–29.0]	27.3 ± 8.7
T11	73.4 [60.7–90.6]	24.8 [21.4–29.6]	29.1 ± 8.6
T12	82.7 [66.4–102.0]	28.1 [23.9–33.3]	29.8 ± 9.2
L1	97.7 [78.9–115.6]	32.7 [28.0–38.5]	30.0 ± 9.4
L2	124.3 [98.5–145.1]	41.5 [34.8–48.3]	29.5 ± 9.1
L3	141.5 [116.8–170.7]	48.5 [41.9–54.8]	30.3 ± 8.8
L4	138.0 [115.5–166.1]	48.0 [41.9–53.6]	30.6 ± 8.8
L5	135.2 [106.6–158.1]	44.8 [38.4–53.5]	34.0 ± 8.5

Data are presented as mean ± SD or as median [interquartile range].

SMA, skeletal muscle area; SMI, skeletal muscle index; MRA, muscle radiation attenuation; HU, Hounsfield units.

The ICC values for the interrater reliability of the SMA and MRA for all vertebra levels ranged from 0.95 to 1.00. All interrater reliability values are shown in Table 3. The power was 1.00.

**TABLE 3:** Intraclass correlation coefficient for interrater reliability.

	SMA		MRA	
	Pearson correlation	Bootstrap [95% interval]	Pearson correlation	Bootstrap [95% interval]
C1	0.96	0.94–0.97	0.98	0.98–0.99
C2	0.98	0.98–0.99	0.99	NA
C3	0.99	0.99–1.00	1.00	NA
C4	1.00	0.99–1.00	1.00	NA
C5	1.00	0.99–1.00	1.00	NA
C6	0.99	0.99–1.00	0.99	NA
C7	0.97	NA	0.98	0.97–0.99
T1	0.97	NA	0.99	NA
T2	0.95	0.93–0.97	0.98	NA
T3	0.95	NA	0.99	NA
T4	0.96	0.92–0.97	0.99	0.98–0.99
T5	0.98	0.96–0.99	0.98	0.96–0.99
T6	0.98	0.97–0.99	1.00	NA
T7	0.99	0.99–1.00	1.00	1.00–1.00
T8	0.99	0.99–1.00	1.00	0.99–1.00
T9	0.99	0.99–0.99	1.00	NA
T10	0.99	0.99–0.99	1.00	NA
T11	0.98	0.98–0.99	0.99	NA
T12	0.98	0.98–0.99	0.99	NA
L1	0.98	0.98–0.99	1.00	NA
L2	0.99	0.98–0.99	1.00	NA
L3	1.00	1.00–1.00	1.00	NA
L4	0.99	0.99–1.00	0.99	NA
L5	0.99	0.99–1.00	1.00	NA

SMA, skeletal muscle area; MRA, muscle radiation attenuation; NA, not appropriate, bootstrap only in case of skewed data.

The Pearson correlation coefficients between the other vertebra levels and L3 are shown in Table 4. All correlations for SMA, SMI, and MRA were statistically significant. For SMA, correlations ranged from *r* = 0.49 to *r* = 0.95. Strong correlations were found between C1–C3 and L3, and C7–L5 and L3 (*r* = 0.72–0.95). For SMI, Pearson correlation coefficients ranged from *r* = 0.49 to *r* = 0.93. Strong correlations were found between the levels C1–C2, C7–T5, T7–L5, and L3 (*r* = 0.70–0.93), respectively. For MRA, the correlation ranged from *r* = 0.48 to *r* = 0.95. Strong correlations were found between T1–L5 and L3 (*r* = 0.71–0.95). The power was 1.00.

**TABLE 4:** Pearson correlation and bootstrap results between other vertebra levels and L3.

L3									
	SMA			SMI			MRA		
	Pearson correlation	*p*-Value	Bootstrap [95% interval]	Pearson correlation	*p*-Value	Bootstrap [95% interval]	Pearson correlation	*p*-Value	Bootstrap [95% interval]
C1	0.77	<0.001	0.70–0.82	0.71	<0.001	0.61–0.78	0.61	<0.001	0.53–0.71
C2	0.77	<0.001	0.71–0.82	0.71	<0.001	0.61–0.79	0.63	<0.001	NA
C3	0.72	<0.001	0.62–0.80	0.67	<0.001	0.53–0.78	0.59	<0.001	NA
C4	0.49	<0.001	0.33–0.65	0.49	<0.001	0.27–0.67	0.58	<0.001	NA
C5	0.53	<0.001	0.42–0.63	0.51	<0.001	0.63–0.64	0.56	<0.001	NA
C6	0.62	<0.001	0.52–0.70	0.57	<0.001	0.45–0.68	0.48	<0.001	NA
C7	0.73	<0.001	NA	0.68	<0.001	NA	0.59	<0.001	0.50–0.70
T1	0.76	<0.001	NA	0.70	<0.001	0.62–0.77	0.71	<0.001	NA
T2	0.77	<0.001	0.70–0.82	0.70	<0.001	0.61–0.77	0.75	<0.001	NA
T3	0.82	<0.001	NA	0.77	<0.001	0.68–0.83	0.79	<0.001	NA
T4	0.86	<0.001	0.82–0.89	0.81	<0.001	0.75–0.86	0.81	<0.001	0.76–0.85
T5	0.79	<0.001	0.74–0.83	0.72	<0.001	0.65–0.78	0.82	<0.001	0.77–0.86
T6	0.74	<0.001	0.69–0.79	0.65	<0.001	0.58–0.72	0.83	<0.001	NA
T7	0.79	<0.001	0.72–0.84	0.71	<0.001	0.62–0.78	0.85	<0.001	0.81–0.89
T8	0.80	<0.001	0.75–0.84	0.73	<0.001	0.70–0.79	0.86	<0.001	0.83–0.90
T9	0.80	<0.001	0.75–0.85	0.74	<0.001	0.67–0.81	0.87	<0.001	NA
T10	0.85	<0.001	0.80–0.89	0.81	<0.001	0.74–0.87	0.87	<0.001	NA
T11	0.91	<0.001	0.88–0.93	0.89	<0.001	0.84–0.92	0.90	<0.001	NA
T12	0.92	<0.001	0.89–0.94	0.90	<0.001	0.85–0.93	0.92	<0.001	NA
L1	0.92	<0.001	0.90–0.94	0.91	<0.001	0.87–0.93	0.93	<0.001	NA
L2	0.95	<0.001	0.93–0.96	0.93	<0.001	0.90–0.95	0.95	<0.001	NA
L4	0.92	<0.001	0.89–0.94	0.89	<0.001	0.84–0.93	0.94	<0.001	NA
L5	0.92	<0.001	0.89–0.93	0.89	<0.001	0.85–091	0.88	<0.001	NA

SMA, skeletal muscle area; SMI, skeletal muscle index; MRA, muscle radiation attenuation; NA, not appropriate, bootstrap only in case of skewed data.

The correlations between the other levels and L3 per tumor localization are shown in Table 5. All *p*-values were significant (*p* ≤ 0.001) and the bootstraps confirmed the correlation values, except for the SMA and SMI at the level of C4–C6 in the patients with esophageal cancer. Level T4 is the uppermost level in the vertebral column that reached a strong correlation with L3 for SMA, SMI, and MRA in all tumor localizations. The power analysis shows that for the smallest group (patients with head and neck cancer, *n* = 34) the power was 0.80 for correlations of *r* = 0.60 and higher. For the largest group (patients with melanoma, *n* = 87) the power was 0.80 for all correlations.

**TABLE 5:** Pearson correlation values according to different tumor localizations.

	Head and neck cancer L3 (*n* = 34)			Esophageal cancer L3 (*n* = 45)			Lung cancer L3 (*n* = 54)			Melanoma L3 (*n* = 87)		
	SMA	SMI	MRA	SMA	SMI	MRA	SMA	SMI	MRA	SMA	SMI	MRA
C1	0.60	0.47	0.87	0.69	0.62	0.64	0.76	0.73	0.76	0.78	0.74	0.35
C2	0.71	0.62	0.86	0.57	0.48	0.80	0.76	0.70	0.74	0.78	0.74	0.39
C3	0.78	0.72	0.89	0.63	0.51	0.70	0.81	0.74	0.77	0.65	0.64	0.30
C4	0.64	0.61	0.74	X	X	0.79	0.70	0.64	0.66	0.44	0.50	0.43
C5	0.47	0.46	0.76	X	X	0.62	0.52	0.45	0.64	0.54	0.57	0.45
C6	0.70	0.63	0.70	X	X	0.49	0.59	0.67	0.46	0.62	0.59	0.42
C7	0.72	0.57	0.71	0.49	0.52	0.61	0.64	0.59	0.53	0.77	0.75	0.56
T1	0.74	0.60	0.82	0.58	0.58	0.74	0.69	0.61	0.81	0.79	0.74	0.60
T2	0.61	0.43	0.85	0.63	0.62	0.74	0.83	0.76	0.84	0.86	0.82	0.66
T3	0.68	0.54	0.83	0.76	0.73	0.80	0.88	0.81	0.87	0.86	0.84	0.74
T4	0.82	0.73	0.85	0.79	0.74	0.80	0.87	0.80	0.87	0.85	0.83	0.77
T5	0.76	0.66	0.88	0.71	0.64	0.84	0.82	0.77	0.86	0.76	0.70	0.76
T6	0.71	0.58	0.88	0.73	0.67	0.88	0.81	0.76	0.87	0.67	0.56	0.78
T7	0.77	0.67	0.91	0.76	0.71	0.89	0.80	0.74	0.88	0.74	0.64	0.79
T8	0.75	0.64	0.91	0.72	0.66	0.85	0.83	0.80	0.88	0.78	0.71	0.84
T9	0.74	0.62	0.91	0.75	0.70	0.87	0.86	0.83	0.90	0.76	0.71	0.82
T10	0.81	0.71	0.90	0.78	0.76	0.92	0.91	0.88	0.90	0.82	0.78	0.83
T11	0.88	0.81	0.93	0.84	0.83	0.91	0.93	0.91	0.92	0.91	0.90	0.86
T12	0.86	0.79	0.94	0.88	0.87	0.93	0.93	0.91	0.95	0.92	0.91	0.89
L1	0.90	0.86	0.95	0.91	0.90	0.92	0.90	0.89	0.96	0.93	0.92	0.91
L2	0.92	0.87	0.97	0.93	0.92	0.93	0.90	0.85	0.97	0.97	0.96	0.92
L4	0.90	0.88	0.98	0.87	0.84	0.93	0.88	0.79	0.95	0.93	0.92	0.92
L5	0.87	0.84	0.94	0.86	0.83	0.88	0.90	0.85	0.85	0.92	0.91	0.87

SMA, skeletal muscle area; SMI, skeletal muscle index; MRA, muscle radiation attenuation; X, bootstrap does not confirm this correlation.

## 4. Discussion

This is the first study to assess the correlation of muscle quantity and quality between all other vertebra levels and L3. For muscle quantity, i.e., SMA and SMI, most cervical, thoracic, and lumbar levels show a strong correlation with L3. Notably, in the group of patients with esophageal cancer, none of the cervical levels correlate strongly with L3 for SMA and SMI. For muscle quality, i.e., MRA, all thoracic and lumbar levels show a strong correlation with the muscle quality of L3, whereas the cervical levels do not. However, in patients with head and neck cancer, all levels, including the cervical, show a strong correlation with muscle quality at the L3 level. Also, in the patients with esophageal and lung cancer, some cervical levels show a strong correlation.

Our findings are in line with previous studies that determined the correlation between other vertebra levels and L3. For example, in patients with head and neck cancer, a strong correlation between the other lumbar levels and L3 was previously found (14, 26, 27). In patients with various types of advanced cancer, only thoracic levels, T5, T8, T10, and T12, have been studied, and moderate correlations for SMI and MRA between T5, T8, T10, and L3 were found (14, 26, 27). In patients with oral squamous cell carcinoma, the correlation between T12 and L3, was strong which is in agreement with the results of our study (28). However, for level C3, results are ambiguous in head and neck cancer patients and C3 was reported to not correlate well with L3 in patients with low muscle mass (29). In contrast, a strong correlation between the muscles at C3 and L3 in patients with head and neck cancer was found in our study. In patients with head and neck cancer, it is more difficult to measure the cervical muscles, because the tumor is located in the cervical region (26). For example, when contouring the sternocleidomastoid muscle, the SMA may be overestimated because the lymph node stations are located around this muscle (30). Doubling the SMA of the healthy sternocleidomastoid muscle to compensate for the lack of the SMA of the affected muscle can be considered, to avoid the muscle quantity being influenced by the tumor at the level of the affected sternocleidomastoid muscle (26). Moreover, a study in patients with head and neck cancer showed no significant difference in the correlation between C3 and L3 when comparing a group of patients with head and neck cancer with healthy participants (26). Unfortunately, we cannot explain why the cervical levels of the patients with esophageal cancer lacked correlation with L3 for muscle quantity. Further research is needed to identify determinants for the this correlation. Cervical MRA values in this study were more homogeneous for patients with melanoma compared to values for patients with other cancer types. This could explain the correlation between cervical levels and L3 being lower in the patients with melanoma compared to the other patients.

Our results confirm excellent interrater reliability of measuring SMA and MRA by CT scan analysis as found in previous research (31–33). Previous research demonstrated that longer time between measurements limits reliability. For example, when participants walk around for a while between the two measurements, the reliability for contouring the SMA was only acceptable (31). In our study, muscle contouring was performed twice on the same CT image. Moreover, the HU values were set and the segmentation was performed semi-automatically. A factor that may influence reliability of MRA is the accuracy of the contouring of the muscles. If intramuscular fat is incorporated in the SMA due to incorrect contouring, this could negatively affect reliability of MRA. In our study, the HU values corrected the contouring of the muscles, to ensure that only muscle tissue was contoured.

The current study has some limitations. Firstly, in 85% of our participants, intravenous contrast was used while taking the PET-CT. Previous research has demonstrated that the use of contrast fluid influences the SMI and MRA (32). More research is needed to determine whether contrast fluid influences the correlation between different vertebral levels (32). Secondly, while we have included a diverse group of patients with cancer with high incidence rates in the Belgian population (33), the sample size for each tumor localization group was small. Moreover, the proportion of women in our study was not large, due to using a convenience sample that reflects the distribution of sex in the patient populations. Therefore, more research with larger sample sizes and equal sex distribution is needed to confirm our conclusions. Thirdly, we were not able to correlate the vertebra levels to whole body muscle mass. Evaluation of whole body muscle mass requires complete inclusion of the arms in the scan, and unfortunately the diameter of the CT scan was set too small, based on the trunk, and therefore did not include the arms.

In the current study, we found that other levels are strongly correlated with L3. However, if a CT scan at the L3 level is not available the other thoracic and lumbar vertebra levels could serve as a proxy to measure muscle quantity in patients with head and neck-, lung-, esophageal cancer, and melanoma, whereas the cervical levels may be less reliable as a proxy in some patient groups. Future research is needed to develop prediction equations to estimate whole body muscle mass from the vertebra levels correlating well with the L3 level.

## 5. Conclusion

In patients with head and neck cancer, lung cancer, and melanoma, muscle quantity is strongly correlated between some cervical, and all thoracic and lumbar levels and L3. In esophageal patients, only the thoracic and lumbar levels are strongly correlated. For muscle quality, the cervical, thoracic, and lumbar levels and L3 are well correlated in the head and neck, esophageal, and lung patients, but in patients with melanoma the cervical levels do not correlate well with L3. If visualization of L3 on the CT scan is absent, we suggest that the other thoracic and lumbar vertebra levels could serve as a proxy to measure muscle quantity in patients with head and neck-, lung-, esophageal cancer, and melanoma, whereas the cervical levels may be less reliable as a proxy in some patient groups. Further research should determine whether our conclusions can be confirmed and that these levels can also be used to estimate whole body muscle mass by examining the correlation of these levels with whole body muscle mass.

## Data availability statement

The raw data supporting the conclusions of this article will be made available by the authors, without undue reservation.

## Ethics statement

The studies involving human participants were reviewed and approved by the Commissie Medische Ethiek Brussel. Written informed consent for participation was not required for this study in accordance with the national legislation and the institutional requirements.

## Author contributions

JV: acquisition, analysis, and interpretation of data, drafting the work, provide approval for publication, and agrees to be accountable for all aspects of the work in ensuring that questions related to the accuracy or integrity of any part of the work are appropriately investigated and resolved. MS, HJ-W, and AS: design of the work, interpretation of data, revising the work, and agrees to be accountable for all aspects of the work in ensuring that questions related to the accuracy or integrity of any part of the work are appropriately investigated and resolved. CB: acquisition of data and revising the work. JK: analysis of data and revising the work. All authors contributed to the article and approved the submitted version.
